# Development and Evaluation of a Simulation-Based Algorithm to Optimize the Planning of Interim Analyses for Clinical Trials in ALS

**DOI:** 10.1212/WNL.0000000000207306

**Published:** 2023-06-06

**Authors:** Jordi W.J. van Unnik, Stavros Nikolakopoulos, Marinus J.C. Eijkemans, Jésus Gonzalez-Bermejo, Gaelle Bruneteau, Capucine Morelot-Panzini, Leonard H. van den Berg, Merit E. Cudkowicz, Christopher J. McDermott, Thomas Similowski, Ruben P.A. van Eijk, for the DiPALS, RespiStimALS and Ceftriaxone Study Group

**Affiliations:** From the Department of Neurology (J.W.J.v.U., L.H.v.d.B., R.P.A.v.E.), UMC Utrecht Brain Center, and Biostatistics & Research Support (S.N., M.J.C.E., R.P.A.v.E.), Julius Center for Health Sciences and Primary Care, University Medical Center Utrecht, the Netherlands; Sorbonne Université (J.G.-B., C.M.-P., T.S.), INSERM, UMRS1158 Neurophysiologie Respiratoire Expérimentale et Clinique, AP-HP, Groupe Hospitalier Universitaire APHP-Sorbonne Université, Site Pitié-Salpêtrière, Département R3S; APHP (G.B.), Groupe Hospitalier Paris 6, Hôpital Pitié-Salpêtrière, Département de Neurologie, Centre Référent SLA, France; Department of Neurology (M.E.C.), Massachusetts General Hospital, Boston; and Sheffield Institute for Translational Neuroscience (C.J.M.), University of Sheffield, United Kingdom.

## Abstract

**Background and Objectives:**

Late-phase clinical trials for neurodegenerative diseases have a low probability of success. In this study, we introduce an algorithm that optimizes the planning of interim analyses for clinical trials in amyotrophic lateral sclerosis (ALS) to better use the time and resources available and minimize the exposure of patients to ineffective or harmful drugs.

**Methods:**

A simulation-based algorithm was developed to determine the optimal interim analysis scheme by integrating prior knowledge about the success rate of ALS clinical trials with drug-specific information obtained in early-phase studies. Interim analysis schemes were optimized by varying the number and timing of interim analyses, together with their decision rules about when to stop a trial. The algorithm was applied retrospectively to 3 clinical trials that investigated the efficacy of diaphragm pacing or ceftriaxone on survival in patients with ALS. Outcomes were additionally compared with conventional interim designs.

**Results:**

We evaluated 183–1,351 unique interim analysis schemes for each trial. Application of the optimal designs correctly established lack of efficacy, would have concluded all studies 1.2–19.4 months earlier (reduction of 4.6%–57.7% in trial duration), and could have reduced the number of randomized patients by 1.7%–58.1%. By means of simulation, we illustrate the efficiency for other treatment scenarios. The optimized interim analysis schemes outperformed conventional interim designs in most scenarios.

**Discussion:**

Our algorithm uses prior knowledge to determine the uncertainty of the expected treatment effect in ALS clinical trials and optimizes the planning of interim analyses. Improving futility monitoring in ALS could minimize the exposure of patients to ineffective or harmful treatments and result in significant ethical and efficiency gains.

Drug development for amyotrophic lateral sclerosis (ALS) has been proven to be difficult. More than 70 therapeutic compounds have been evaluated over the past 30 years,^1-3^ resulting in only minimal gains in life expectancy^4^ or amelioration of progression rates.^5,6^ Significantly, several interventions have led to an accelerated loss of function and reduction of survival time,^7-11^ emphasizing that trial participation may not always be free from harm. The high futility rate among ALS clinical trials, together with the risks associated for patients, stresses the importance—both ethically and economically—of developing more efficient strategies, to identify ineffective or harmful treatments more quickly and thereby improve the safety of trial participants and minimize the loss of time, funding, and resources.

Currently, most ALS clinical trials perform their statistical analyses after all patients have completed a fixed follow-up period.^12,13^ As an alternative, interim analyses could be performed on the accumulating data when only a subset of patients have completed their follow-up.^14^ This presents the opportunity to stop a trial before all patients have been enrolled, or completed the study, as soon as there is sufficient evidence for a drug's (in)efficacy. The probability that a trial can be stopped early, however, depends strongly on the timing and the prespecified decision rules or stopping criteria.^15^ These criteria are often defined arbitrarily at the design stage of a study and frequently ignore information that is available from earlier clinical trials.^16^ As a result, the planning and stopping criteria of interim analyses may be suboptimal, which could potentially mean that ineffective treatments are continued or exposure to placebo is prolonged unnecessarily.^12^

In this study, we develop a simulation-based algorithm that determines the optimal timing of interim analyses, together with their decision rules, by incorporating information obtained in earlier stages of development, and minimize the expected trial duration or sample size. By applying the algorithm to 3 completed clinical trials, we aim to evaluate its performance and obtain insights into optimization strategies to better tailor study designs for future clinical trials.

## Methods

This study consisted of 2 parts: first, we developed a simulation-based algorithm to determine the optimal timing and decision rules for interim analyses, on the basis of prior knowledge. Second, we applied the algorithm retrospectively to 3 clinical trials that investigated the efficacy and safety of diaphragm pacing or ceftriaxone in patients with ALS. We evaluated whether the studies could have been stopped earlier, and when, and compared the results with other commonly used interim analysis schemes or designs without interim analyses.

### Interim Analyses for Clinical Trials

During an interim analysis, using the accumulating data, one determines whether there is sufficient evidence to stop the study early for either drug efficacy (superiority) or lack of it (futility) or whether it is better to continue the trial and collect more information. If interim analyses are used, the investigator must prespecify: (1) the timing of the analyses (i.e., after how many months/patients/events) and (2) the decision rules about when a treatment is considered (in)effective. These decision rules are statistical criteria that define how large the (standardized) effect size or how small the *p* value should be to stop the trial. As such, interim analyses result in multiple statistical tests, requiring a correction of the *p* value for the treatment effect. There are several ways to adjust *p* values, for example, by using a Pocock design^17^ that sets the same significance threshold for all interim analyses uniformly (e.g., stop if *p* < 0.01). Or, alternatively, one can use the more common O'Brien-Fleming design^18^ that uses stringent significance thresholds at early interim analyses (e.g., stop if *p* < 0.001), but sets more lenient significance thresholds at later interim analyses (e.g., stop if *p* < 0.045). The benefit of the Pocock design is that it becomes easier to stop at early interim analyses and, on average, results in shorter studies. The downside is that it becomes more difficult to stop at later interim analyses if the trial cannot be stopped early. Consequently, such an interim analysis scheme potentially requires more patients or a longer duration compared with an O'Brien-Fleming design. As such, prespecifying the interim analyses at the design stage of any trial is crucial. By varying the number of interim analyses, together with their timing and decision rules, many unique interim analysis schemes can be defined with different probabilities of stopping early and expected trial durations. The scheme that one will consider to be most efficient depends primarily on the treatment effect that one expects.^16^

### Uncertainty About the Expected Treatment Effect

The expected treatment effect, therefore, is a decisive assumption at the design stage of any trial, which will drive the efficiency of an interim analysis scheme. The expected treatment effect is usually based on either drug-specific information obtained in earlier development stages (e.g., small phase 2 studies) or defined arbitrarily. In many instances, however, there is a considerable degree of uncertainty around the expected treatment effect.^19^ In addition, especially for ALS, the success rate of any clinical development program is low, and overall, most trials result in a futile conclusion.^20^ Ideally, the probability of success and the overall uncertainty about the treatment effect need to be accounted for to find the optimal interim analysis scheme, given the information available at the design stage.

### Optimization Algorithm for Interim Analyses

**Figure 1:**
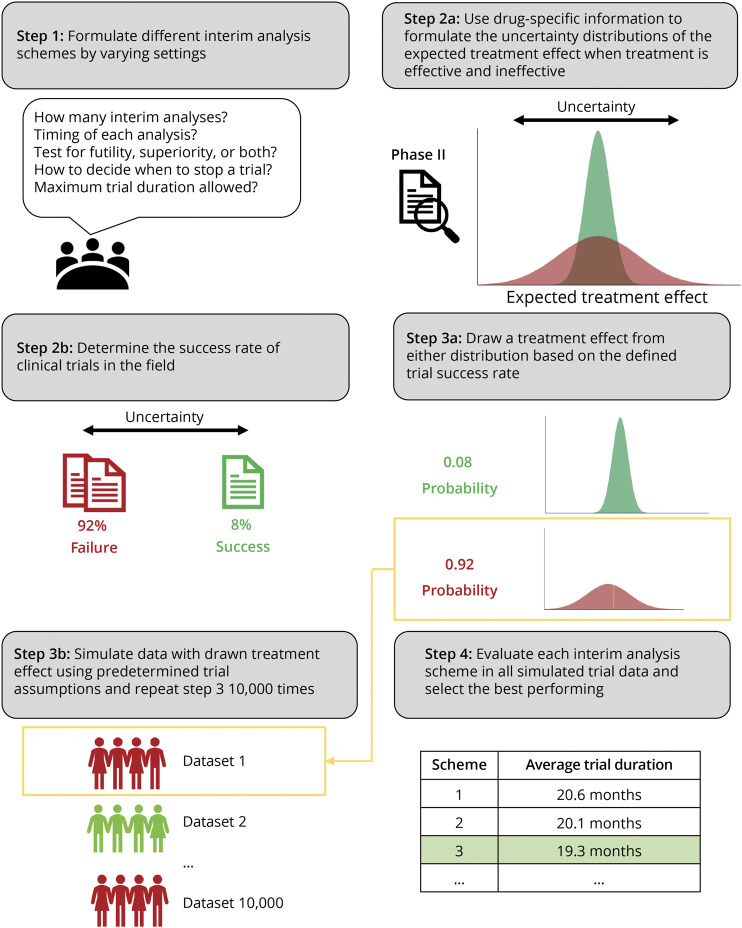
Schematic Illustration of the Simulation-Based Algorithm to Optimize Interim Analysis Schemes First, the investigators decide on the range of acceptable interim analysis schemes. Previous research is then used to formulate the uncertainty around the treatment effect and the probability of trial success. In the next step, each interim analysis scheme is evaluated by means of simulation based on the trial assumptions made. Finally, the best performing interim analysis scheme is identified, based on a selection criterion (e.g., shortest average trial duration, smallest average sample size, or shortest average drug exposure). A script to replicate the simulation is provided in eAppendix 1 (links.lww.com/WNL/C768).

We approached this challenge by means of simulation, thereby resampling the treatment effect from a distribution reflective of both the uncertainty in the treatment effect and the overall probability of success. For each simulation, we compared different interim analysis schemes and selected the design with the most efficient operating characteristics (e.g., trial duration, randomized patients, or drug exposure). Figure 1 provides a schematic of our optimization process. First, we defined all possible interim analysis schemes by varying the timing, number of analyses, and decision rules (step 1). Second, we defined 2 uncertainty distributions for the treatment effect: (1) for the scenario that treatment is effective and (2) for the scenario that treatment is ineffective (step 2a). Next, we defined the probability that a trial will be successful (step 2b). Finally, we sampled a treatment effect from our uncertainty distribution, conditional on whether the trial would be successful or not (step 3a). The drawn treatment effect was subsequently used to simulate a clinical trial dataset (step 3b), in which the different interim analysis schemes were evaluated (Figure 1, step 4).

### Application of Algorithm to ALS Clinical Trials

We applied the abovementioned algorithm retrospectively to 3 completed ALS clinical trials (DiPALS, RespiStimALS, and Ceftriaxone-ALS) to evaluate whether using an optimized interim analysis scheme could have concluded the studies earlier. The study population and methods of the trials are described elsewhere.^10,11,21^ In short, all trials were randomized controlled clinical trials that assessed the efficacy and safety of diaphragm pacing (DiPALS and RespiStimALS) or ceftriaxone (Ceftriaxone-ALS) in patients with ALS. Patients in the DiPALS study were randomized to receive either noninvasive ventilation (NIV) plus diaphragm pacing or NIV alone, whereas patients in the RespiStimALS study received either diaphragm or sham pacing. All patients receiving (sham) pacing were operated laparoscopically. The primary endpoint of the DiPALS study was time to death, whereas the RespiStimALS study used time to death or NIV. For the Ceftriaxone-ALS study, patients were randomized to receive either intravenous ceftriaxone or placebo. This study used a coprimary endpoint assessing both time to death or respiratory insufficiency and the change in daily function (Revised ALS Function Rating Scale [ALSFRS-R]). To harmonize the 3 clinical trials, we used only the survival endpoint of the Cetriaxone-ALS study. While DiPALS and RespiStimALS studies were originally designed without interim analyses, the Ceftriaxone-ALS trial had already planned 5 interim analyses. The DiPALS and RespiStimALS trials were stopped prematurely because of safety issues resulting in excess deaths in the pacing arms, whereas the Ceftriaxone-ALS trial was stopped early for ineffectiveness. All patients provided informed consent, and all studies were approved by an appropriate ethical committee.

Both pacing trials were designed based on an unpublished, historically controlled, multicenter cohort study that led to Humanitarian Device Exemption approval of diaphragm pacing by the US Food and Drug Administration.^22^ In that report, the median survival of the historical cohort who received usual care was 21.4 months (n = 43), whereas the median survival in the cohort who received pacing was 37.5 months (n = 43). To define the uncertainty around the expected treatment effect, we assumed that the cohort was followed up for 24 months and that survival time followed a Weibull distribution (ρ = 2),^23^ resulting in a standard error of the log-hazard ratio (HR) of 0.335.^24^ Significantly, the success rate of any phase 3 clinical trial in ALS is 7.1% (2 successes of 28 trials).^20^ As such, one should be even more uncertain about the expected treatment effect. To inflate the uncertainty around the expected treatment effect, we defined a mixture distribution, sampling the standard error of the log-HR from a β distribution with a 0.071 probability of 0.335 and a 1 − 0.071 probability of 1.06 (i.e., 10 times the variance of the historical data). To illustrate, if we were to design a trial with a HR of 0.45, the 75% uncertainty level around that estimate, conditional on the success probability and historical data, ranges from 0.23 to 0.87. For the ceftriaxone trial, we assumed a median survival of 28.9 months for patients receiving placebo and a median survival of 43.4 months for those receiving ceftriaxone. Based on the observed functional decline of the ALSFRS-R in the phase II trial,^25^ and assuming that every point increase in ALSFRS-R reduces the hazard for death by 11.9%,^26^ we estimated the standard error of the log-HR for ceftriaxone to be 0.147. We then defined the mixture distribution similar as described earlier. Based on the assumptions defined in the respective trial protocol (Table 1), we simulated clinical trial data for each study and determined their optimal interim analysis scheme.

**Table 1:**
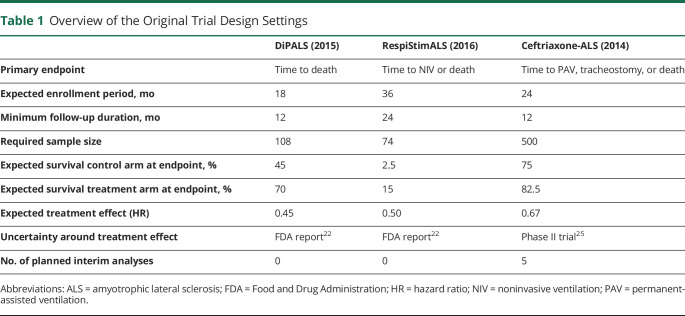
Overview of the Original Trial Design Settings

### Statistical Analysis

Time-to-event data were simulated using a Weibull distribution assuming a constant enrollment rate and no loss to follow-up for the primary endpoint. A log-rank test was used to compare treatment arms after a certain number of events had been reached (i.e., an event-driven trial design).^27^ In each simulated dataset, we evaluated a variety of interim analysis schemes. These varied in number of futility analyses from 4 to 5 and in timing ranging from 30% to 90% of the maximum number of events, with steps of 10%. In all schemes, the interim analysis for superiority was fixed at 60% because stopping early for efficacy is often undesirable.^28,29^ The nonbinding futility boundary ranged from Pocock type to O'Brien-Fleming type (i.e., β-spending function from 0.75 to 3.00 with steps of 0.25), whereas the superiority boundary ranged from 2.00 to 3.00.^30^ Schemes that had a maximum trial duration of more than 10% compared with a design without interim analyses were excluded.^31^ Clinical trial scenarios were simulated 10,000 times. The interim testing scheme with the shortest average trial duration was selected as the most optimal design. In addition, we applied 4 conventional interim designs to the 3 completed ALS clinical trials to compare the performance of the optimized interim analysis schemes to other design approaches. The number of futility analyses in these conventional interim designs varied from 1 to 4 at arbitrarily defined time points using O'Brien-Fleming–type decision rules.^18^ Finally, to investigate alternative treatment effects, we simulated the treatment effect, from very beneficial to very harmful, in each of 3 trials while keeping enrollment rate, survival in the placebo arm, and randomization ratio fixed. For each scenario, we determined when the trial could be stopped, using designs either with or without (optimized) interim analyses. We highlight the comparison of the optimized interim analysis schemes with designs without interim analyses or with a single interim analysis at 60% of the maximum number of events, which reflects commonly used interim design settings.^32^ All statistical analyses were performed with the R language for statistical programming and the package *Rpact* (version 3.2.2; Wassmer G and Pahlke F, 2022); the script to replicate the simulations is provided in eAppendix 1 (links.lww.com/WNL/C768).

### Data Availability

Investigators may request access to the de-identified individual participant data from the respective corresponding authors of the DiPALS and RespiStimALS studies or from the National Institute of Neurological Disorders and Stroke for access to the Ceftriaxone-ALS data.

## Results

Based on information available at the design stage, we evaluated 797 interim analysis schemes for the DiPALS trial, 183 schemes for the RespiStimALS trial, and 1,351 schemes for the Ceftriaxone-ALS trial. The number of eligible schemes varied between trials due to differences in the original design settings and their implications for the maximum trial duration. For the DiPALS trial, the optimal interim analysis scheme consisted of 4 interim analyses at 30%, 40%, 50%, and 60% of the (maximum) planned number of events (α-spending and β-spending parameters: 2.50). For the RespiStimALS trial, the optimal scheme consisted of 4 interim analyses at 30%, 50%, 60%, and 70% (α-spending and β-spending parameters of 2.25 and 2.75, respectively). For the Ceftriaxone-ALS trial, the optimal scheme consisted of 4 interim analyses at 30%, 40%, 50%, and 60% (α-spending and β-spending parameters: 2.25).

### Application of Optimized Interim Analysis Scheme

**Figure 2:**
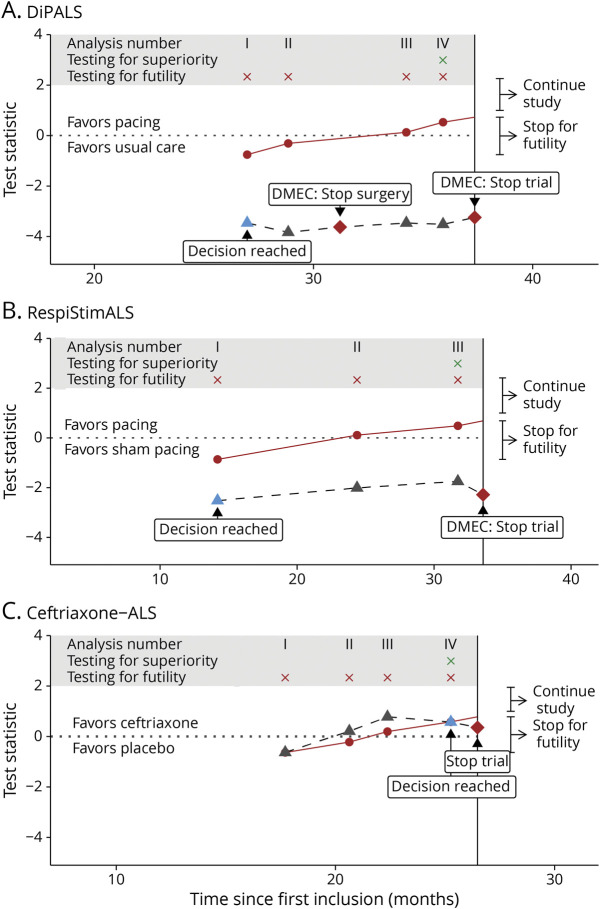
Retrospective Application of the Algorithm in the 3 ALS Clinical Trials Performance of the optimized interim analysis schemes when applied retrospectively to the DiPALS (A), RespiStimALS (B), and Ceftriaxone-ALS (C) trials. The black dashed line reflects the development of the test statistic over time, which is calculated sequentially and represents the treatment evidence based on the data accumulated thus far. As soon as the treatment evidence surpasses the red line, the trial can be stopped for futility. This line, therefore, represents the decision rules of the trial. In addition, a horizontal dotted line was plotted to indicate whether treatment evidence favors treatment or control. The blue triangle represents the stopping decision of the optimized interim analysis scheme, whereas the red diamond reflects when the trial was actually stopped. ALS = amyotrophic lateral sclerosis; DMEC = Data Monitoring and Ethics Committee.

The DiPALS trial was discontinued prematurely by the Data Monitoring and Ethics Committee (DMEC). The stop decision is illustrated in Figure 2A together with the test statistic for the treatment effect at each time point. In addition, we provide the decision boundaries for the optimized interim analysis scheme. As can be seen, the observed test statistic for the treatment effect at the first interim analysis is lower than the futility boundary. As such, the trial could have been stopped for futility at that time, leading to reduction of 34.0% in trial duration, and could have reduced the number of randomized patients by 17.6%.

**Figure 3:**
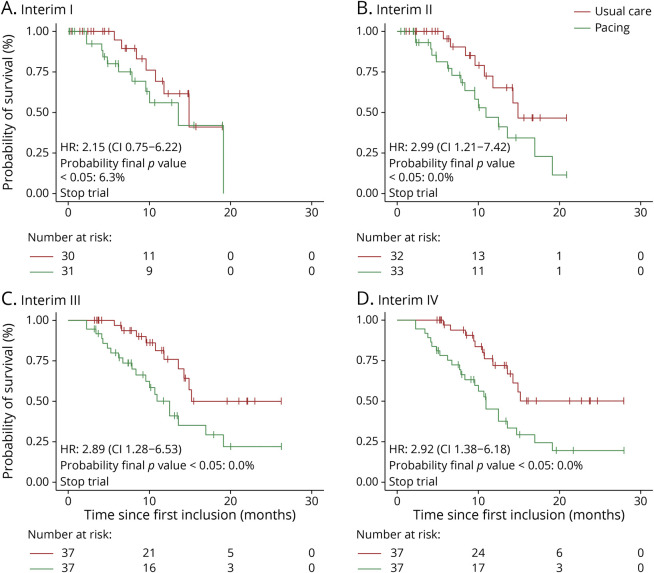
Development of the Primary Outcome in the DiPALS Trial Development of the observed treatment effect over time at each interim analysis (A–D). For illustrative purposes, we show the development of the Kaplan-Meier curves at each interim analysis of the DiPALS trial. At the first interim analysis (A), the hazard ratio (HR) is 2.15 (0.75–6.22), and the trial can be stopped (Figure 2A). Taking into account the accumulated data at this time, and the expected HR of 0.45, the probability that the final analysis may still yield statistical significance in favor of treatment is only 6.3%.

A comparison with the original design is detailed in Table 2 and with a nonoptimized interim analysis scheme in Table 3. In Figure 3, we illustrate the maturity of the survival curve at each interim analysis for the DiPALS study, together with the probability of reaching statistical significance if the trial had continued, conditional on the expected treatment effect at the design stage. For example, at the first interim analysis, we observed an HR of 2.15 in favor of usual care. The probability, if we were to continue the trial and collect more data, of this treatment effect shifting from an HR of 2.15 toward statistical significance in favor of treatment, is 6.3%. As can be seen, this so-called “conditional power” approaches zero at subsequent interim analyses, effectively ruling out the possibility that the trial will be successful.

**Table 2:**
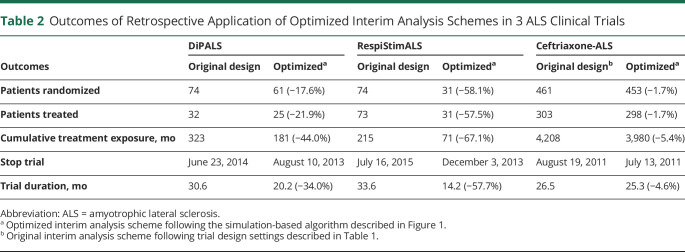
Outcomes of Retrospective Application of Optimized Interim Analysis Schemes in 3 ALS Clinical Trials

**Table 3:**
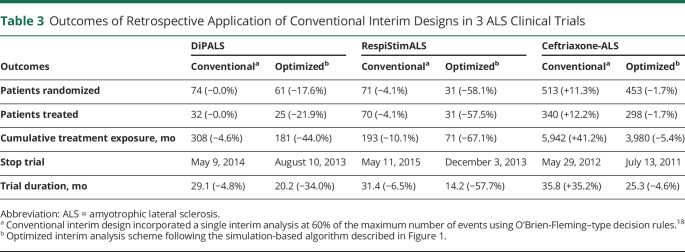
Outcomes of Retrospective Application of Conventional Interim Designs in 3 ALS Clinical Trials

Similarly, Figure 2, B and C, presents the decisions for the RespiStimALS and Ceftriaxone-ALS trials. In both cases, the optimized interim analysis scheme could reach a conclusion before the original design, leading to a reduction in trial duration of 57.7% for RespiStimALS and of 4.6% for Ceftriaxone-ALS, and could have reduced the number of randomized patients by 58.1% and 1.7%, respectively (Table 2). In both cases, a conventional interim design would be less efficient (Table 3). For other conventional interim designs, we provide an overview of the results for each trial in eTable 1 (links.lww.com/WNL/C770). In all 3 trials, each interim analysis scheme would outperform a design without interim analyses.

**Figure 4:**
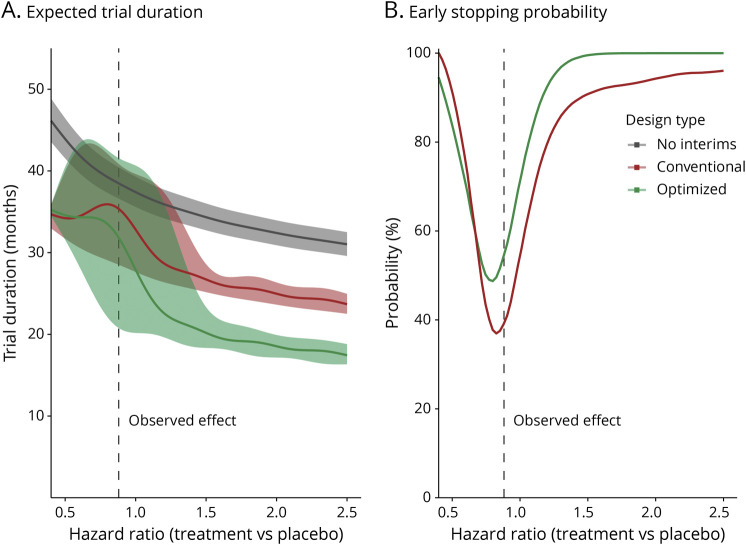
Behavior of the Optimized Interim Analysis Scheme Under Different Treatment Effects for Ceftriaxone We simulated the effect of ceftriaxone from very beneficial to very harmful (10,000 simulations per scenario), while keeping the randomization ratio, enrollment rate, and survival in the placebo arm fixed. For each scenario, we determined when the trial could be stopped, using the optimized interim analysis scheme (green), a conventional interim design (red), and a design without interim analyses (gray). An interim analysis scheme is most effective when there is a large positive or negative treatment effect when compared with a design without interim analyses (A). In addition, we illustrated the probability that a trial conclusion would result in a more than 20% reduction in trial duration, using the optimized (green) and conventional (red) interim designs vs a design without interim analyses (B). Conventional interim design incorporated a single interim analysis at 60% of the maximum number of events using O'Brien-Fleming–type decision rules.^18^

Finally, in Figure 4, we present the behavior of the optimized interim analysis scheme under different treatment effects for ceftriaxone. Likewise, we report the results for diaphragm pacing in eFigure 1 (links.lww.com/WNL/C769). As can be seen, the optimized scheme is most effective when there is a large positive or negative treatment effect in reducing trial duration when compared with a design without interim analyses. In eTable 2 (links.lww.com/WNL/C771), we report the results of other conventional interim designs had the trial concluded a neutral treatment effect (i.e., HR = 1). For each trial, the optimized interim scheme would, on average, be the most efficient design.

## Discussion

In this study, we have explored an optimization strategy for the planning of interim analyses in clinical trials. As opposed to using arbitrary design settings, our simulation-based algorithm uses information from earlier clinical trials in combination with historical success probabilities to optimize the study design. Objective selection of the most optimal monitoring scheme can then be based on the preferred operational characteristics, such as the expected trial duration, number of patients, or cumulative drug exposure time. By retrospective application of our algorithm to 3 completed ALS trials, we have shown that optimized interim analysis schemes may lead to considerable reductions in trial duration and number of patients randomized when compared with designs without interim analyses or with arbitrarily defined interim analysis schemes. Consequently, the time of patient exposure to ineffective or harmful treatments can be minimized, and resources may be reallocated sooner to other promising therapeutic candidates.

Considering the high futility rates in ALS clinical trials, the pressing unmet medical need, and the ever-increasing pipeline of potential pharmaceutical agents,^3^ it is imperative to identify (in)effective or harmful treatments quickly. This is further underlined by the conduct of several trials in the past, which have led to accelerated loss of function and reduction in survival time compared with usual care.^7-11^ Interim analyses have been widely reported to be beneficial,^33^ a conclusion supported by our findings. Although the use of such analyses has been the subject of requests from the patient community and consensus guidelines,^34,35^ surprisingly, they have not been implemented very frequently in ALS trials.^12,13^ Common barriers may include lack of expertise and experience, increased effort required in the planning and conduct of analyses, insufficient funding to compensate for lack of design familiarity, and the preference for more familiar methods.^36^ Even when interim analyses are implemented, their planning and decision rules are often based on arbitrary and subjective choices. This is undesirable because the timing of interim analyses, and their stopping rules, have a significant impact on the study efficiency and probability to stop early.^16^ Our algorithm resolves this challenge by enabling an objective and data-driven approach to the planning of interim analyses and their decision rules, while not increasing the complexity of the study.

The optimal interim analysis scheme depends primarily on the treatment effect that one expects to observe.^16^ The uncertainty around the expected treatment effect is typically assumed to be zero, which may affect the trial if the actual treatment effect is considerably different. This risk can be mitigated by formulating an uncertainty distribution around the expected treatment effect.^19^ For example, if there is no evidence of any therapeutic benefit at the design stage, or if the success rate of clinical development is low, one may want to focus primarily on futility, while one would be more focused on stopping early for efficacy if a previous trial had been successful. Our algorithm automates such considerations and assesses the ideal trade-off between early stopping for futility and/or efficacy based on the uncertainty in the treatment effect at the design stage. In some settings, however, stopping early for efficacy could be undesirable, and limiting the number of superiority analyses may be warranted.^28,29^ We have accommodated this by implementing asymmetric testing schemes that solely test for futility at early interim analyses and only evaluate efficacy after a certain amount of information has been collected.

The amount of information required should be evaluated on a case-by-case basis and depends on the development phase of the trial. One consideration, for example, includes whether the results would be sufficiently persuasive for regulators if the trial were stopped at the interim analysis. Moreover, in some settings, aggressive futility monitoring carries the risk of terminating a trial early that might have demonstrated a beneficial effect had it been continued. This risk, however, does not differ from a design without futility monitoring because these error probabilities are fixed from the outset and equal to a design without interim analyses. An important exception to the rule includes a study in which treatment has a delayed effect on the outcome.^37^ In such instances, very early futility monitoring may increase the possibility of drawing the wrong conclusion. When planning early interim analyses, therefore, the trade-off between early stopping risks, the gain in efficiency, and the minimum required level of information should be considered carefully.

In our study, we considered only early trial termination. However, additional strategies may be of interest to further advance the benefit of interim analyses, such as sample size reestimation, adjustment of the randomization ratio, or enriching the study through amended eligibility criteria. Others have shown how such modifications can positively affect the conduct and efficiency of clinical trials clinical trials^38,39^ and could be valuable extensions of our proposed algorithm. Especially, the modification of eligibility criteria could be of particular relevance for a heterogeneous disease such as ALS, where some patients could benefit more from treatment than others.^40^ Prospectively implementing interim analyses to enrich the trial population may help to identify responding subgroups earlier and may overcome the need to run additional confirmative studies. These efforts may be further improved by using historical data during the interim analysis, for example, within a Bayesian framework, and borrow information to reduce sample sizes or improve precision,^41,42^ or making better use of prediction rules to improve the identification of subgroups.^43,44^ Of importance, these endeavors should be further expanded to key intermediate outcomes, such as the ALSFRS-R and vital capacity, to increase generalizability of our findings, for example, by retrospective application in clinical trials with beneficial effects, such as edaravone, sodium phenylbutyrate-taurursodiol, or methylcobalim.^5,45,46^

Our study has a few limitations. First, we assumed that an interim analysis would be conducted as soon as the target number of events is reached. This is, however, overoptimistic because there is usually a delay between the date the event is reached and the actual analysis. In practice, therefore, the efficiency of the interim analysis scheme could be maximized by ensuring efficient communication between trial staff and the DMEC and by optimizing data flow.^47^ Nevertheless, the amount of time required to complete an analysis may remain considerable, meaning it is not feasible to conduct 4 or more interim analyses for some settings. The algorithm, however, can be easily adjusted to limit the candidate interim analysis schemes to solely 2 or 3 analyses. Our algorithm may be further improved by also considering other sources of uncertainty, such as the uncertainty in the hazard rate, expected enrollment rate, and dropout percentage. Although these variables have no impact on the statistical power if an event-driven design is used,^27^ they do affect the expected trial duration and could result in different optimal monitoring schemes. Open-access initiatives, such as Answer ALS and Pooled Resource Open-Access ALS Clinical Trials, could act as key source to model these sources of uncertainty in the natural history of ALS.^48,49^

In conclusion, the pressing unmet medical need and high failure rate of clinical trials in ALS stresses the urgency—both ethically and economically—to reform the design of studies and explore more efficient alternatives. We have proposed a strategy that aims to tailor the study design based on the available information, a priori, to not only discard futile and harmful treatments early, but also stop the study soon if there is sufficient evidence for effectiveness. The application of our optimization strategy in future ALS clinical trials may, therefore, not only minimize the exposure of patients to ineffective or harmful drugs, but also accelerate the search for beneficial treatments for this devastating disease.

## Glossary

ALS: amyotrophic lateral sclerosis
ALSFRS-R: Revised ALS Function Rating Scale
DMEC: Data Monitoring and Ethics Committee
HR: hazard ratio
NIV: noninvasive ventilation
